# Deficient Sumoylation of Yeast 2-Micron Plasmid Proteins Rep1 and Rep2 Associated with Their Loss from the Plasmid-Partitioning Locus and Impaired Plasmid Inheritance

**DOI:** 10.1371/journal.pone.0060384

**Published:** 2013-03-28

**Authors:** Jordan B. Pinder, Mary E. McQuaid, Melanie J. Dobson

**Affiliations:** Department of Biochemistry and Molecular Biology, Dalhousie University, Halifax, Nova Scotia, Canada; Université de Sherbrooke, Canada

## Abstract

The 2-micron plasmid of the budding yeast *Saccharomyces cerevisiae* encodes copy-number amplification and partitioning systems that enable the plasmid to persist despite conferring no advantage to its host. Plasmid partitioning requires interaction of the plasmid Rep1 and Rep2 proteins with each other and with the plasmid-partitioning locus *STB*. Here we demonstrate that Rep1 stability is reduced in the absence of Rep2, and that both Rep proteins are sumoylated. Lysine-to-arginine substitutions in Rep1 and Rep2 that inhibited their sumoylation perturbed plasmid inheritance without affecting Rep protein stability or two-hybrid interaction between Rep1 and Rep2. One-hybrid and chromatin immunoprecipitation assays revealed that Rep1 was required for efficient retention of Rep2 at *STB* and that sumoylation-deficient mutants of Rep1 and Rep2 were impaired for association with *STB.* The normal co-localization of both Rep proteins with the punctate nuclear plasmid foci was also lost when Rep1 was sumoylation-deficient. The correlation of Rep protein sumoylation status with plasmid-partitioning locus association suggests a theme common to eukaryotic chromosome segregation proteins, sumoylated forms of which are found enriched at centromeres, and between the yeast 2-micron plasmid and viral episomes that depend on sumoylation of their maintenance proteins for persistence in their hosts.

## Introduction

The 2 µm plasmid is a benign parasitic plasmid harbored in the nucleus of most strains of the budding yeast, *Saccharomyces cerevisiae* (For review see [1] and [2]). Despite providing no selective advantage to the host, the plasmid is faithfully maintained at ∼60 copies per cell [3]. The 2 µm plasmid achieves this by encoding copy-number amplification and partitioning systems [1], [2], and by borrowing host cell machinery for its replication [4] and segregation [5]–[9]. Retention of the 2 µm plasmid at normal copy number is also dependent on the host cell process of sumoylation [10]–[13], the post-translational modification of target proteins with the small ubiquitin-related modifier protein SUMO [14].

Sumoylation is an essential conserved eukaryotic function known to regulate diverse cellular processes by modulating the interactions, localization, or post-translational stability of substrate proteins (reviewed in [14]–[16]). Like ubiquitin, SUMO must be activated in a series of enzyme-catalyzed steps before being conjugated to target proteins by the E2 conjugating enzyme Ubc9. Some target proteins require an E3 ligase [17], [18] for recruitment to Ubc9. In yeast, two SUMO-specific deconjugating enzymes, Ulp1 and Ulp2, remove SUMO conjugates from distinct subsets of target proteins [19], [20]. Ulp1 also processes the SUMO primary translation product to its mature form. Combined loss of the SUMO E3 ligases Siz1 and Siz2 [11], loss of the SUMO-targeted ubiquitin-ligase Slx5-Slx8 [13], or mutation or mislocalization of Ulp1 [10], [12] has been found to lead to elevated levels of the 2 µm plasmid, indicating that plasmid maintenance is dependent on host cell sumoylation.

Amplification of the plasmid copy number is mediated by the plasmid site-specific recombinase, Flp, and in wild type yeast occurs only when the copy number drops below the normal level [21], [22]. Flp is directly targeted for sumoylation [11]. SUMO conjugates have also been observed for one of the two plasmid-encoded partitioning proteins, Rep2, and suggested for the other, Rep1 [11], [12]. When Flp sumoylation is impaired, an aberrant high-molecular weight form of the plasmid accumulates [23], demonstrating that Flp function is normally regulated by this host cell post-translational modification. We have previously observed mis-segregation of a fluorescently-tagged 2 µm plasmid in yeast defective for the SUMO-specific protease Ulp1 [12], suggesting that plasmid partitioning might be regulated by sumoylation, but the effect of Rep protein sumoylation on plasmid maintenance has not been investigated.

Equal partitioning of the multiple copies of the 2 µm plasmid during mitosis depends on interaction of Rep1 and Rep2 with each other, and with a tandemly-repeated sequence at the plasmid *STB* locus ([24] and reviewed in [2] and [25]). Mutations in Rep1 that impair these interactions [24], or absence of any one of these three elements results in most newly budded daughter cells failing to receive copies of the plasmid [26]. Association of Rep1 and Rep2 with *STB* aggregates the multiple copies of the plasmid into a small number of nuclear foci that persist throughout the cell cycle [5], [27], and localize near spindle pole bodies [5], [7]. Equal partitioning of these clustered plasmids between the mother and daughter cell at mitosis is dependent on conversion of the *STB* chromatin to a form that shares some features with centromeres. The *STB*-associated Rep proteins recruit the kinesin-related motor protein Kip1 [8] which is required for exchange of the canonical histone H3 in the *STB*-bound nucleosome for the centromere-specific histone H3 variant Cse4 [9], for association of the RSC2 chromatin remodeling complex [24], [28], [29], and for recruitment of the cohesin complex to *STB* [6], [24], a process that is also dependent on the integrity of the mitotic spindle [7]. The precise mechanism by which the Rep proteins perform these functions is unknown. Here we investigate the contribution of sumoylation of both Rep1 and Rep2 to plasmid inheritance. We demonstrate that mutations in Rep1 and Rep2 that impair sumoylation also impede their stable association with *STB*, and plasmid inheritance, suggesting similarity to the sumoylation-dependent localization of eukaryotic host chromosome segregation proteins to centromeres, and to viral episomes that depend on sumoylation of their maintenance proteins for faithful persistence in the host.

## Experimental Procedures

### Yeast strains and media

Standard methods were used for growth and manipulation of yeast and bacteria [30], [31]. Yeast were cultured in YPAD (1% yeast extract, 2% Bacto Peptone, 0.003% adenine, 2% glucose), synthetic defined (0.67% Difco yeast nitrogen base without amino acids, 2% glucose, 0.003% adenine, 0.002% uracil, and all required amino acids), or synthetic complete medium (0.67% Difco yeast nitrogen base without amino acids, 2% glucose, 1% Difco casamino acids, 0.003% adenine, 0.002% uracil, 0.002% tryptophan) [30]. For induction of *GAL* promoters, glucose was replaced with galactose (2%). Media were supplemented with 200 µg/mL geneticin (G418) (Sigma) and 100 µg/mL nourseothricin (clonNAT) (Werner BioAgents) for selection of *KanMX6-* and *NatMX6*-tagged gene replacements, respectively. Yeast were transformed by the LiAC/SS-DNA/PEG method [32].

Yeast strains used in this study are given in Table 1. Strains lacking the 2 µm circle, designated *cir* ^0^, were derived from strains containing the 2 µm circle, *cir^+^*, by expression of a defective Flp recombinase from the plasmid pBIS-GALkFLP-(TRP1) [33]. Yeast gene deletion strains were created by targeted replacement of wild-type alleles with *KanMX6* gene deletion alleles amplified by PCR from appropriate strains in the EUROSCARF yeast gene deletion strain collection [34] using recommended primers and conditions. Transformed yeast were selected for G418-resistance and gene deletions confirmed by PCR. *NatMX6*-tagged deletion strains were derived from the corresponding *KanMX6*-tagged deletion strains by targeted gene replacement.

**Table 1:** Yeast strains used in this study.

Name	Relevant genotype	Reference or parental strain
W303a/*α* [*cir* ^+^]	*MAT* ***a*** */MATα ade2-1/ade2-1 ura3-1/ura3-1 leu2-3,112/leu2-3,112 his3-11his3-11,15 trp1-1,trp1-1 [cir^+^]*	[79]
W303a/*α* [*cir* ^0^]	*MAT* ***a*** */MATα ade2-1/ade2-1 ura3-1/ura3-1 leu2-3,112/leu2-3,112 his3-11/his3-11,15 trp1-1,trp1-1 [cir^0^]*	W303a/*α* [*cir* ^+^]
W303/1a	*MAT* ***a*** * ade2-1 ura3-1 leu2-3,112 his3-11,15 trp1-1 [cir^+^]*	[79]
MD83/1b	*MAT* ***a*** * ade2-1 ura3-1 leu2-3,112 his3-11,15 trp1-1 [cir^0^]*	[12]
MD83/1c	*MATα ade2-1 ura3-1 leu2-3,112 his3-11,15 trp1-1 [cir^0^]*	[12]
JP91/4	*MATα smtΔ:KanMX6 [pRS426-CUP1p-HA_3_-SMT3(GG)] [cir^0^]*	this study
CTY10/5d	*MAT* ***a*** * gal4 gal80 his3-200 trp1-901 ade2 ura3-52 leu2-3,112 met thr URA3:(lexAop)_8_-lacZ [cir^+^]*	[80]
CTMD/3a	*MAT* ***a*** * his3 trp1 leu2-3,112 ade2-1 ura3 met URA3:(lexAop)_8_-lacZ*	this study
MD83/29	*MAT* ***a*** * GFP-lacI:HIS3 [cir^0^]*	this study
EP4	*MAT* ***a*** * gal4 gal80 his3-200 trp1-901 ade2 ura3-52 leu2-3.112 met thr URA3:STB-p-HIS3 [cir^+^]*	CTY10/5d
EP4MD [*cir* ^+^]	*MAT* ***a/*** *MATα gal4/GAL4 gal80/GAL80 his3-11,-15/his3-200 trp1-1/trp1-901 ade2-1/ade2 ura3-1/ura3-52 leu2-3,-112/leu2-3,-112 MET/met THR/thr URA3: STB-p-HIS3 [cir^+^]*	this study
EP4MD [*cir* ^0^]	*MAT* ***a/*** *MATα gal4/GAL4 gal80/GAL80 his3-11,-15/his3-200 trp1-1/trp1-901 ade2-1/ade2 ura3-1/ura3-52 leu2-3,-112/leu2-3,-112 MET/met THR/thr URA3: STB-p-HIS3 [cir^0^]*	EP4MD [*cir* ^+^]
EGY48 [*cir* ^+^]	*MATα ura3 his3 trp1 (lexAop)_6_:LEU2 [cir^+^]*	[81]
EGY48 [*cir* ^0^]	*MATα ura3 his3 trp1 (lexAop)_6_:LEU2 [cir^0^]*	EGY48 [*cir* ^+^]
JP98/2	*MAT* ***a*** * ade2-1 ura3-1 leu2-3,112 his3-11,15 trp1-1 rsc2Δ:NatMX6 [cir^0^]*	MD83/1b
IRS10	*MAT* ***a*** * pAS10ΔORI:ADE2 [cir^0^]*	MD83/1b
IRS133	*MAT* ***a*** * pAS-rep1_3R_ΔORI:ADE2 [cir^0^]*	MD83/1b
IRS135	*MAT* ***a*** * pAS-rep2_13R_ΔORI:ADE2 [cir^0^]*	MD83/1b

### One-hybrid and two-hybrid assay expression plasmids

pGAD424- and pSH2-1-derived plasmids expressing Rep1, Rep2, or mature yeast SUMO fused to the Gal4 transcriptional activation domain (Gal4_AD_) or to the bacterial LexA repressor protein, respectively, have been previously described [12]. The genomic DNA encoding the Nis1 SUMO interaction-motif-containing domain [35] inserted in plasmid pSH-NIS1_357–408_ was isolated in a library screen (unpublished data). A mutant version of *REP1* encoding an I202T substitution (*REP1_I202T_*) was isolated in screen for mutagenized *REP* genes unable to maintain a 2 µm-based plasmid (unpublished data). The *REP1*_I202T_ open reading frame (ORF) was amplified by PCR and inserted in pGAD424 and pSH2-1 to create pGAD-REP1_I202T_ and pSH-REP1_I202T_, respectively, as previously described for the wild type *REP1* ORF [36]. *TRP1* gene-based plasmids to direct galactose-inducible expression of the Rep proteins as HA-epitope-tagged B42 transcriptional activation domain (B42_AD-_HA)-fusions in yeast and that were either 2 µm-based (pMM2) or single-copy *CEN/ARS* (pMM3) were created by digestion of pJG4-5 [37] with BamHI and NotI. The overhangs were made flush and the plasmid self-ligated to create pMM1. A 6.1-kb EcoRI/SphI fragment from pMM1 was ligated with EcoRI/SphI fragments from pGAD424, pGAD-REP1, pGAD-REP2 [36], and pGAD- REP1_I202T_, to yield pMM2, pMM2-REP1, pMM2-REP2, and pMM2- REP1_I202T_, respectively. The 2 µm backbone in the pMM2-based plasmids was removed by digestion with KpnI and EagI and then replaced with KpnI/EagI-digested pRS314 [38] to generate the single copy *CEN/ARS* plasmids pMM3-based versions of these plasmids. All PCR-amplified genes were checked after cloning by sequencing. Plasmid pGAD-SUMOΔGG was created by subcloning a SmaI/PstI fragment from pGBD-*smt3ΔGG* [35] (a generous gift from M. Hochstrasser) encoding conjugation-defective SUMO, into pGAD424.

### 2 µm-based plasmids

Plasmid pAS4, a *flp^-^ ADE2*-tagged version of the 2 µm circle that can be propagated in yeast and *E. coli*, has been previously described [36]. The *ADE2* gene insertion disrupts the *FLP* gene, and the Flp target site between the *REP1* and *REP2* genes has been deleted and replaced with the *E.coli* vector pTZ18R (Pharmacia). pAS10 is identical to pAS4 but with pTZ18R inserted in the opposite orientation. Site-directed mutagenesis of *REP1* and *REP2* was carried out by gap repair of plasmids pAS10 and pAS4, respectively (primer sequences are available upon request). PCR amplicons containing either the *REP1* ORF flanked by ∼650 bp upstream and ∼450 bp downstream or the *REP2* ORF flanked by ∼900 bp upstream and ∼600 bp downstream and containing the designated point mutation(s) were created by assembly PCR, and co-transformed into yeast with NruI/SalI-digested pAS10 and SphI-digested pAS4, respectively. Plasmids were isolated in *E. coli*, mutations confirmed by sequencing, and plasmids re-transformed into yeast for subsequent experiments. In order to combine various *REP1* and *REP2* alleles in a single *ADE2 flp^-^* 2 µm plasmid, a PCR product encoding from ∼900 bp upstream of the *REP2* ORF to ∼980 bp into the *REP1* ORF in a pAS4-based plasmid was used for gap repair of BamHI/SphI-digested pAS10-based plasmids. *ADE2*-tagged 2 µm plasmids directing expression of Ubc9- or His_6_-N-terminally-tagged Rep1 and Rep2 proteins expressed from their own promoters were created by assembly PCR and gap repair in a similar fashion to plasmids encoding point-mutant *REP* alleles. Ubc9 fusion proteins contained five glycine residues between the carboxy-terminal lysine residue in Ubc9 and the first residue of Rep1 or Rep2. To create *ADE2*-tagged 2 µm-based plasmids lacking the *REP* genes, pAS4 was digested with SphI and the plasmid self-ligated to make pAS4*ΔREP2*, and an XhoI site was introduced upstream of the *REP1* coding region in pAS10 by PCR, and the resulting plasmid digested with XhoI and SalI and self-ligated to create pAS10*ΔREP1*. To create pKan4 in which the *ADE2* gene in pAS4 is replaced with *KanMX6*, yeast harboring pAS4 were transformed to G418-resistance with a PCR product encoding the *ade2Δ:KanMX6* allele. pKan4 was isolated in *E. coli* and re-transformed into yeast for all subsequent experiments. An identical strategy was used to derive all other *KanMX6*-tagged 2 µm plasmids from pAS4/pAS10-based plasmids. To create yeast strains containing the *REP1* and *REP2* genes with their flanking 2 µm sequences chromosomally-integrated at the *ADE2* locus, pAS4 was digested with BclI and SnaBI to remove the origin of replication, the ends were made flush and the plasmids self-ligated to create pAS10ΔORI. pAS10ΔORI was linearized with NdeI and used to transform *cir* ^0^ strains to adenine prototrophy.

### Other plasmids

To create *TRP1*- or *LEU2*-based *CEN/ARS* plasmids for expression of untagged Rep proteins from the *GAL1* promoter, the *REP1* and *REP2* ORFs, encoding wild-type or mutant Rep1 and Rep2 alleles, were amplified by PCR and cloned into plasmids pRSGAL-TRP and pRSGAL-LEU, which were created by replacement of the PvuII fragment encoding the multiple cloning site of pRS314 and pRS315 [38], respectively, with the 1.4-kb PvuII fragment from pESC-URA (Stratagene) containing the *GAL1* promoter.

Plasmids for galactose-inducible expression of untagged (pJP2-*SMT3*) and HA-tagged (pJP2-HA-*SMT3*) SUMO were constructed by using assembly PCR to synthesize products encoding untagged or HA-tagged mature SUMO that were used to gap repair pMM2, with deletion of the B42_AD_-HA coding region.

The *URA3*-based integrative one-hybrid vector pJL638 was a kind gift from J. Li [39]. The integrative one-hybrid *HIS3* reporter gene plasmid pEP1 was created from pJL638 by replacing the MscI/SstI fragment encoding the *lacZ* ORF, which lies downstream of a basal yeast promoter, with an MscI/SstI fragment encoding the *HIS3* gene ORF with its transcription termination sequences. The repeated sequence from the 2 µm *STB* locus (a 295-bp AvaI/HpaI fragment) was converted to an XhoI fragment by addition of linkers and inserted at the XhoI site upstream of the *HIS3* reporter gene in pEP1 to create pEP1-STB. *STB* one-hybrid *HIS3* reporter strains were created by using StuI-linearized pEP1-STB to transform yeast to uracil prototophy.

### One-hybrid and two-hybrid assays

To test for interaction of the Rep proteins with *STB*, the one-hybrid reporter strain EP4MD was transformed to tryptophan prototrophy with pMM2- or pMM3-based plasmids encoding various Rep1 and Rep2 alleles. Transformants were grown overnight in selective liquid medium containing glucose, and were serially diluted, spotted on solid medium containing galactose, and imaged after seven days incubation at 28°C. For two-hybrid assays testing for interaction of the Rep proteins with each other and with SUMO, co-transformants in the *cir* ^0^ two-hybrid reporter strain CTMD/3a were assayed for β-galactosidase expression by a filter assay. Specificity of interactions was confirmed by co-expressing LexA-fusion proteins with the Gal4_AD_ or Gal4_AD_-Snf4, and Gal4_AD_-fusion proteins with LexA [40]. Two-hybrid interactions were also assayed in the *cir* ^0^ reporter yeast strain EGY48 by spotting serial dilutions of co-transformants onto solid medium lacking leucine to assess expression of the *LEU2* reporter gene.

### Plasmid loss assays

To determine the rate of loss of *KanMX6*-tagged 2 µm plasmids, yeast transformants were initially grown in YPAD medium containing the antibiotic G418 to select for retention of the plasmid. The proportion of plasmid-containing cells was then determined both before and after ∼15 generations of growth in medium that did not select for the retention of the plasmid (YPAD) by comparing plating efficiency on solid YPAD medium containing or lacking G418. Statistical significance was determined using an unpaired t test.

### Protein analysis

For all applications protein was extracted from yeast by alkaline lysis as previously described [11], [41]. Briefly, ∼10^8^ cells were pelleted, resuspended in 200 µL of lysis solution (1.85 M NaOH, 7.4% β-mercaptoethanol) and incubated for 10 min on ice. Protein was precipitated for 10 min by addition of 200 µL cold 50% trichloroacetic acid, pelleted by centrifugation, washed twice with 1 mL cold acetone and thoroughly dried.

For western blot analysis, protein was resuspended in equal volumes of urea extraction buffer (8 M urea, 100 mM NaH_2_PO_4_/Na_2_HPO_4_, 50 mM Tris) and 2× protein gel loading buffer (125 mM Tris pH 6.8, 4.0% SDS, 20% glycerol, 4.0% β-mercaptoethanol, 1 M urea, 0.05% bromophenol blue, 0.05% xylene cyanol). Protein suspensions were briefly vortexed, centrifuged at 16 000× *g* for 1 min and supernatants analyzed as previously described [36]. Primary antibodies were rabbit-derived polyclonal anti-Rep1 or anti-Rep2 [36], and mouse anti-Pgk1 (Molecular Probes), or mouse anti-HA (Sigma). Secondary antibodies for chemiluminescent detection were horseradish peroxidase (HRP)-conjugated goat anti-rabbit and anti-mouse IgG (KPL), and for fluorescent detection were anti-mouse Dylight 488, anti-mouse Dylight 549, and anti-rabbit Dylight 649 (Rockland). Chemiluminescence was generated using an ImmunStar Western C kit (BioRad) and captured either by X-ray film or digitally using a charge-coupled device (CCD) camera in a VersaDoc 4000 MP imaging system (BioRad). Fluorescence was digitally captured using the VersaDoc 4000 MP system equipped with the appropriate LED/filter combination.

For metal ion affinity chromatography, dried protein pellets were resuspended in 1 mL of binding buffer (6 M guanidine HCl, 300 mM NaCl, 50 mM sodium phosphate, pH 8.0) supplemented with 10 mM N-ethylmaleimide, vortexed briefly, and clarified by centrifugation at 16 000× *g* for 15 min. Supernatants were transferred to a microfuge tube containing ∼20 µL TALON resin (Clontech) and rocked at room temperature for 2 h. Resin was briefly washed three times with wash buffer (8 M urea, 300 mM NaCl, 50 mM sodium phosphate, 5 mM imidazole, pH 7.5), and then 4 µL of 1.5 M imidazole was added, followed by 25 µL of 2× protein gel loading buffer. Resin suspensions were boiled for 5 min, clarified by centrifugation and ∼5 µL analyzed by western blotting.

### Chromatin immunoprecipitation (ChIP)

For ChIP analysis of Rep protein association with the plasmid *STB* locus, 2 µm-based plasmids tagged with an *ADE2* adenine biosynthetic gene were used. The *ADE2*-tagged plasmids are maintained at higher copy number than the equivalent *KanMX6*-tagged plasmids when yeast are grown under conditions selecting for the presence of the plasmids, with a copy number closer to that of the native 2 µm plasmid. ChIP assays were performed essentially as described [9], [42] with the following modifications. Yeast cultures (50 mL) were grown to OD_600_ ∼1–2 and fixed with 1% formaldehyde for 15 min at room temperature. Crosslinking was quenched by addition of 125 mM glycine and after 5 min cells were harvested, washed with water three times and resuspended in 400 µL cold lysis buffer D (50 mM HEPES-KOH pH 7.5, 140 mM NaCl, 1 mM EDTA, 1% Triton X-100, 0.1% SDS, 1× complete protease inhibitor cocktail (Roche)). Yeast were kept chilled for the following steps. Cells were lysed using glass beads, and chromatin was sheared to 0.1–1 kbp fragments by 8 rounds of sonication for 12 s each using a Branson 250 sonifier set to an output level of 3 and 50% duty cycle. Sonicated lysates were clarified by centrifugation at 16 000× *g* for 15 min, the supernatant transferred to a new tube, centrifuged again at 16 000× *g* for 10 min, and 20–100 µL of supernatant (whole cell extract) brought up to 400 µL with lysis buffer D, and incubated for 4–16 h with polyclonal anti-Rep1, anti-Rep2, or monoclonal anti-FLAG (Sigma) antibodies at 4 °C. Protein A Sepharose CL-4B (GE Healthcare) was blocked with 20 µg of sonicated salmon sperm DNA and 100 µg bovine serum albumin for ≥ 1 h, and 20 µL of beads added to the immunoprecipitation mixtures and incubated for 1 h. Beads were washed six times at room temperature as follows: twice with 1 mL ChIP wash buffer I (50 mM Hepes-KOH, pH 7.5, 150 mM NaCl, 1 mM EDTA, 0.1% sodium deoxycholate, and 1% Triton X-100) for 5 min each, twice with 1 ml of ChIP wash buffer II (50 mM Hepes-KOH, pH 7.5, 500 mM NaCl, 1 mM EDTA, 0.1% sodium deoxycholate, and 1% Triton X-100) for 5 min each, once with 1 ml of ChIP wash buffer III (10 mM Tris-HCl, pH 8.0, 250 mM LiCl, 1 mM EDTA, 0.5% sodium deoxycholate, and 0.5% NP-40) for 5 min, and once with 1 mL of TE for 5 min. Chromatin was eluted in SDS, decrosslinked, digested with proteinase K, and DNA extracted with phenol and chloroform as described [30]. DNA was resuspended in 100 µL of TE, and serial dilutions of template DNA were amplified by 30 cycles of PCR with primers flanking *STB*, which were 5′- ATTATAGAGCGCACAAAGGAGA-3′ and 5′- TGCACTTCAATAGCATATCTTTG -3′. PCR products were resolved by agarose gel electrophoresis, stained with ethidium bromide, imaged using a VersaDoc MP 4000 imaging system (BioRad) and quantified by densitometry using QuantityOne software (BioRad). Specificity of co-immunoprecipitation was assessed by comparing the amount of *STB* DNA immunoprecipitated by anti-Rep1 and anti-Rep2 antibodies to that pulled down by the anti-FLAG antibody, and by assessing the degree of non-specific immunoprecipitation of a chromosomal target (*CEN3*) (not shown).

### Fluorescence microscopy

All images were digitally captured using a Nikon80i fluorescent microscope with a Nikon DS-Qi1Mc digital camera and processed using NIS-Elements Basic Research software. Immunofluorescence analysis of yeast was carried out essentially as described [30]. Logarithmically-growing yeast were fixed in 3.7% formaldehyde for 1 h, digested with zymolyase 20T (ICN), and spotted to Superfrost Plus slides (Fisher). After 30 min the fixed spheroplasts were dried in cold methanol for 6 min and cold acetone for 30 s, and rehydrated in blocking buffer (PBS with 2% BSA). Slides were incubated at 4°C overnight with affinity-purified anti-Rep1 or anti-Rep2 antibodies diluted in blocking buffer. Slides were washed twice in PBS, incubated at room temperature for 1 h with 1∶500 AlexaFluor594-conjugated goat anti-rabbit secondary antibody (Invitrogen) in blocking buffer, and washed twice in PBS. Mounting media containing 100 ng/mL 4′,6-diamidino-2-phenylindole (DAPI) was added prior to imaging. To visualize the localization of the *TRP1*-marked 2 µm reporter plasmid pSV5 containing the 2 µm origin of replication, *STB*, and 256 lac operator sequences [6], a GFP-LacI repressor fusion protein was expressed from a chromosomally-integrated gene as previously described [5]. All images are representative of at least two independent experiments in which >200 cells were scored.

## Results

### Multiple lysine substitutions in Rep1 and Rep2 are required to abolish their two-hybrid interaction with SUMO

**Figure 1:**
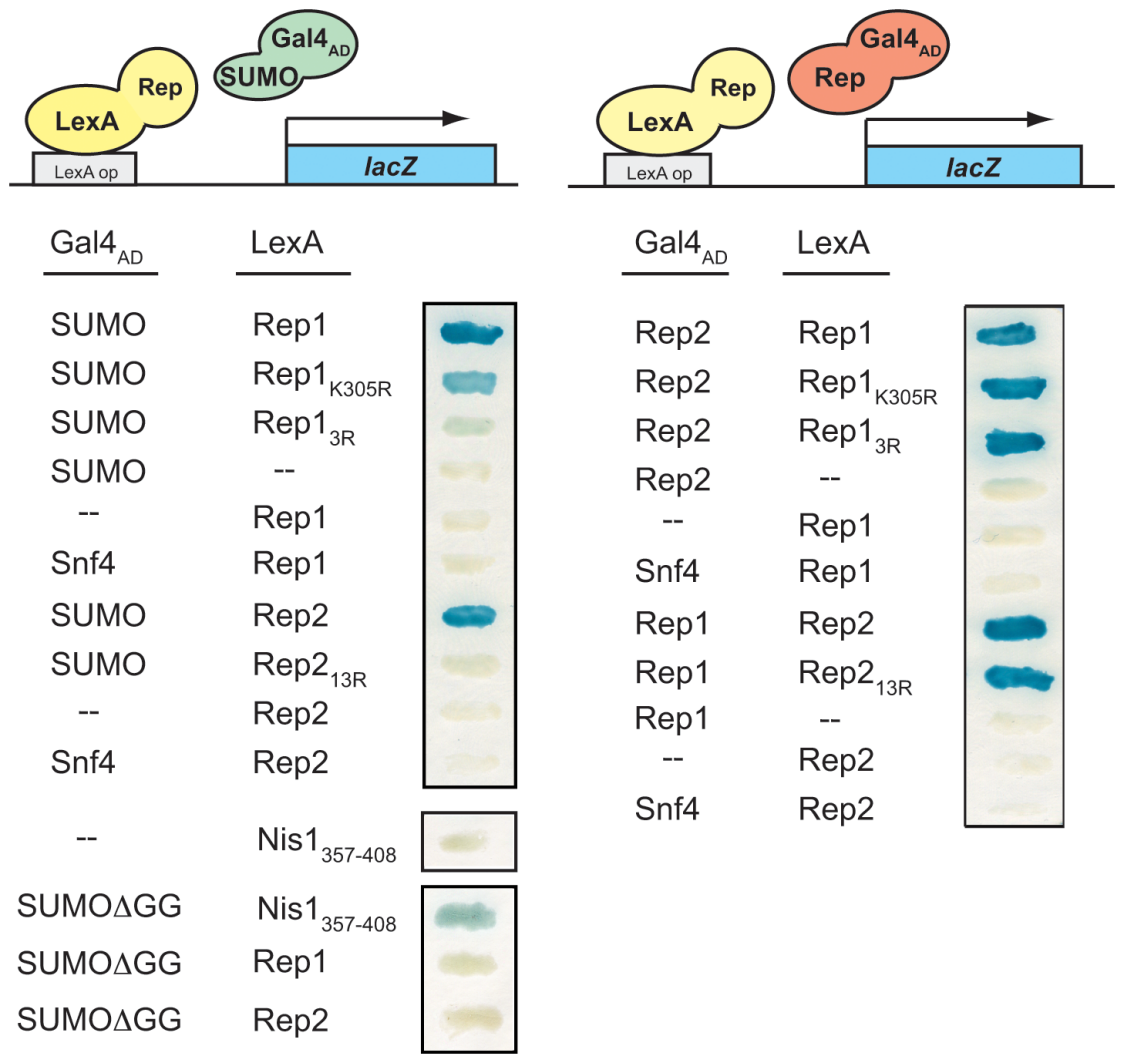
Lysine mutations in Rep1 and Rep2 impair two-hybrid interaction with SUMO but do not affect Rep1-Rep2 interaction. A *cir^0^* two-hybrid reporter yeast strain was co-transformed with plasmids expressing the indicated Gal4_AD_ and LexA fusion proteins. Co-transformants were grown for 24 h on a nitrocellulose membrane and interaction of the fusion proteins was assessed by monitoring expression of the *lacZ* reporter gene using a β-galactosidase filter assay with the substrate X-gal, which produces a blue precipitate upon cleavage. Vector with no insert is indicated (--). Assays for interaction with conjugation-competent SUMO and conjugation-defective SUMOΔGG are shown on left and those for interaction between Rep1 and Rep2 on right.

We have previously observed that both 2 µm plasmid partitioning proteins, Rep1 and Rep2, interact with SUMO *in vivo* in a two-hybrid assay, suggesting they might be direct targets of sumoylation [12]. SUMO-conjugated forms of Rep2 have been observed [11], while sumoylated forms of Rep1 have not been definitively identified to date. Although both Rep proteins might be directly targeted for sumoylation, a two-hybrid interaction would also be observed if either Rep protein could interact with SUMO indirectly. To determine whether the two-hybrid association with SUMO reflected non-covalent recognition of SUMO through a SUMO-interaction motif (SIM), both proteins were tested for their ability to interact with a truncated version of SUMO lacking the two C-terminal glycine residues required for covalent attachment (SUMOΔGG) [35]. Neither Rep1 nor Rep2 interacted with SUMOΔGG in a two-hybrid assay, while a fusion protein containing the previously characterized SIM domain from the yeast Nis1 protein [35] did display interaction with this truncated form of SUMO (Fig. 1, bottom left). The lack of interaction of the Rep proteins with SUMOΔGG indicates that neither Rep protein contains a SIM, consistent with the results of *in vitro* protein interaction assays in which SUMO did not bind to *E. coli*-expressed Rep1 or Rep2 (unpublished data).

Although the two-hybrid association of the Rep proteins with SUMO could be due to interaction of Rep1 and Rep2 with a host protein that is conjugated to SUMO, previously reported observations of SUMO-conjugated forms of Rep2 [11] suggested that at least for Rep2, the two-hybrid interaction with SUMO might result from covalent modification. A mutational approach was undertaken based on the premise that mutation of lysine residues in Rep1 and Rep2 normally targeted for sumoylation should lead to loss of the two-hybrid interaction with SUMO if the interaction solely reflected covalent SUMO modification. We first identified potential sumoylation sites in Rep1 and Rep2. Rep2 lacks a canonical sumoylation motif, (I/V/L)-K-x-(E/D) [43], while Rep1 contains one consensus site at K348. Rep1 and Rep2 mutants containing one or more lysine-to-arginine substitutions were created by site-directed mutagenesis and tested for two-hybrid interaction with SUMO in a yeast reporter strain that lacked the native 2 µm plasmid (*cir* ^0^) to exclude endogenous Rep proteins from participating in the interactions. No single mutation, including Rep1-K348R, completely abolished two-hybrid association with SUMO for either protein, indicating that target lysines were in non-canonical sites and suggesting that more than one lysine residue could be targeted for sumoylation in both Rep1 and Rep2 (Tables 2 and 3, and Fig. 1, top left). The two-hybrid interaction of Rep1 with SUMO was partially reduced for the Rep1-K305R mutant, suggesting this might be a major SUMO acceptor site, and was almost completely abolished in the triple lysine mutant Rep1-K305,315,328R (Rep1_3R_), consistent with these three sites being direct targets for covalent addition of SUMO. Similarly, although different combinations of multiple lysine-to-arginine substitutions in Rep2 slightly reduced two-hybrid interaction with SUMO, simultaneous mutation of thirteen lysine residues in Rep2-K42,44,92,124,130,134,146,148,149,177,208,226,227R (Rep2_13R_) was required to virtually eliminate the two-hybrid interaction with SUMO (unpublished data and Fig. 1). Western blot analysis established that the loss of interaction in our assay was not due to a reduction in steady-state levels of the respective mutant Rep fusion proteins (unpublished data). The mutant Rep proteins also retained their normal two-hybrid interaction with each other (Fig. 1, right) suggesting that the proteins were not grossly misfolded. Taken together, the data suggest that the loss of two-hybrid interaction between the Rep1_3R_ and Rep2_13R_ mutant proteins and SUMO might be due to loss of the lysine residues normally targeted for sumoylation.

**Table 2:** Two-hybrid interactions of Rep1 alleles with SUMO.

Rep1 allele	SUMO interaction
WT	+++
K11, 44, 45, 47, 68, 105, 117, 125, 131, 146, 159, 169, 190, 204, 212, 261, 290, 295, 297, 305, 315, 328, 348R	−
K11, 44, 45, 47, 68, 105, 117, 125R	+++
K146, 159, 169, 190, 204, 212, 261, 290, 295, 297, 305, 315, 328, 348R	−
K146, 159, 169, 190, 204, 212R	+++
K290, 295, 297, 305, 315, 328, 348R	−
K290, 295, 297, 305, 315R	+
K305, 315, 328, 348R	−
K305, 315, 328R ( = **3R**)	−
K305, 315, 348R	+
K305, 315R	+
K305R	++
K315R	+++
K328R	+++

**Table 3:** Two-hybrid interactions of Rep2 alleles with SUMO.

Rep2 allele	SUMO interaction
WT	+++
K8, 13R	+++
K42, 44R	+++
K92R	+++
K95R	+++
K124R	+++
K130R	+++
K134R	+++
K146, 148, 149R	++
K158R	+++
K177R	+++
K208R	+++
K226, 227R	+++
K42, 44, 92, 124, 130, 134, 146, 148, 149, 177, 208, 226, 227R ( = **13R**)	−

### Lysine substitutions in the Rep1_3R_ and Rep2_13R_ mutants impair conjugation to SUMO

If the lysine-to-arginine mutations in Rep1_3R_ and Rep2_13R_ that led to loss of interaction with SUMO in the two-hybrid assay impaired sumoylation, we would expect a reduction in their respective sumoylated forms. While we did observe slower-migrating species of wild-type Rep1 or Rep2 by western blot analysis of protein extracted from yeast containing the native 2 µm plasmid, these species were often barely detectable above background, probably due to the relatively low steady-state levels of the native Rep proteins (unpublished data) and consistent with previously reported observations that Rep1 SUMO conjugates could not be definitively detected, even after Rep1 was affinity-purified [11].

**Figure 2:**
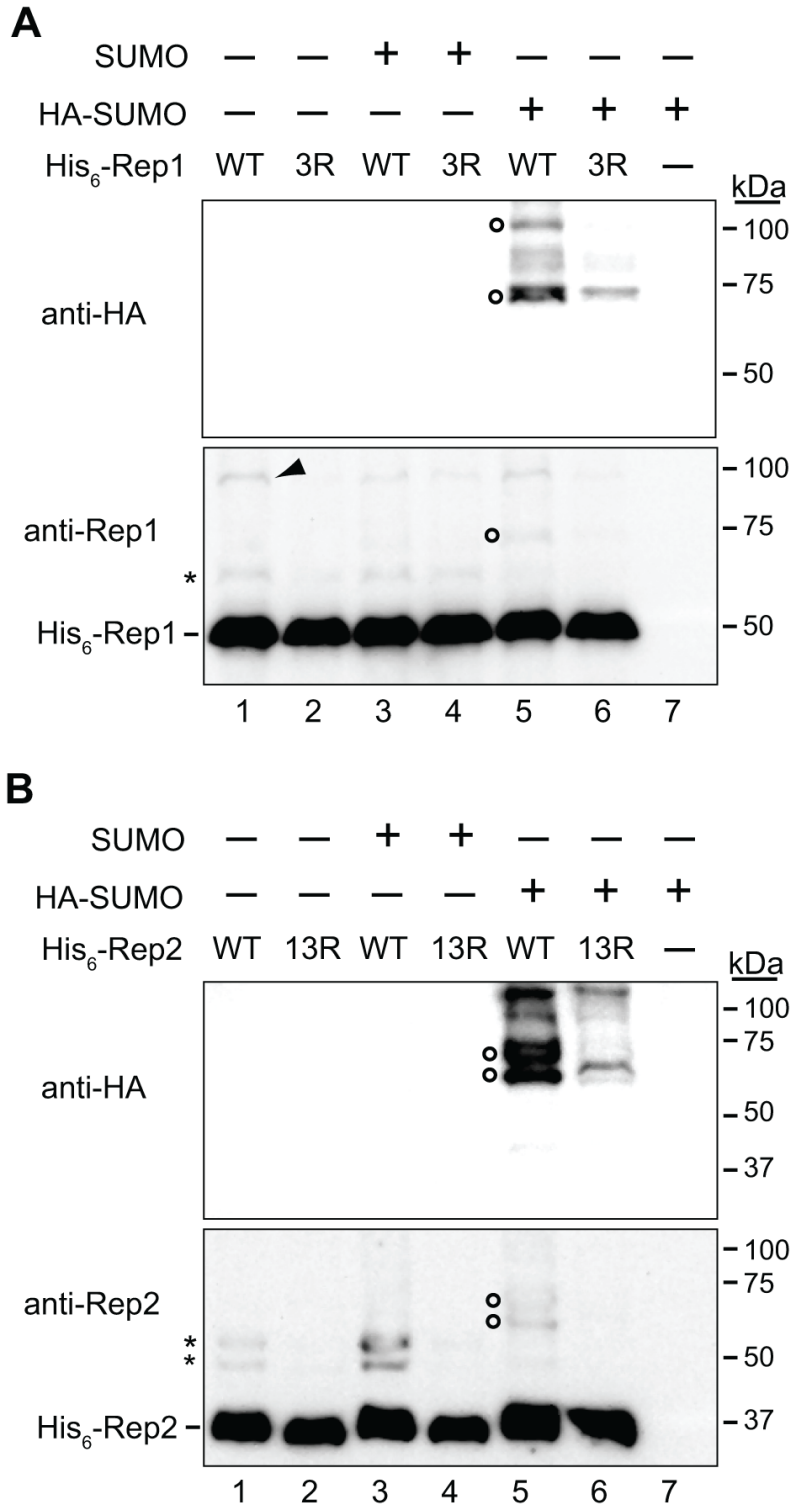
Lysine-to-arginine mutations significantly reduce levels of SUMO-conjugated Rep1 and Rep2. Yeast lacking the native 2 µm plasmid were co-transformed with a plasmid expressing untagged SUMO, HA-epitope-tagged SUMO (HA-SUMO), or no protein (-) from a galactose-inducible promoter, and an *ADE2*-tagged 2 µm plasmid encoding (A) Ubc9-tagged Rep2 and His_6_-tagged Rep1 (wild-type (WT) (lanes 1, 3 and 5), or Rep1_3R_ (lanes 2, 4 and 6)) or (B) Ubc9-tagged Rep1 and His_6_-tagged Rep2 (wild type (WT) (lanes 1, 3, and 5) or Rep2_13R_ (lanes 2, 4, and 6)). Yeast were also transformed solely with the plasmid expressing HA-epitope-tagged SUMO (A and B, lane 7). Yeast were cultured in medium containing galactose for 20 h to induce expression of SUMO proteins. Protein was extracted and His_6_-tagged proteins were affinity purified with Co^2+^ resin and analyzed by western blotting with anti-Rep1, anti-Rep2, or anti-HA antibodies. Species consistent with SUMO-conjugated (asterisks) and HA-SUMO-conjugated (open circles) forms of Rep1 and Rep2, and an unknown Rep1 species (arrowhead) are indicated. Sizes of molecular weight standards resolved in the same gels are indicated.

To artificially enhance the stoichiometry of Rep protein sumoylation, Rep1 and Rep2 were expressed as fusions with the SUMO-conjugating enzyme Ubc9. Ubc9 fusion-directed sumoylation (UFDS) is typically used to enhance auto-sumoylation of the Ubc9-chimera [44]–[46]. However, because Ubc9 itself is sumoylated [35], it would be difficult to discern whether a SUMO conjugate of the Ubc9-Rep fusion protein represented SUMO-modification of the Ubc9 moiety rather than of Rep1 or Rep2. Because Rep1 and Rep2 directly interact *in vivo*, we tested whether fusion of Ubc9 to Rep1 could enhance sumoylation of Rep2 and vice-versa. Yeast were transformed with marker-tagged 2 µm plasmids encoding one Rep protein fused with Ubc9, and the other fused to a hexahistidine tag. Total protein was extracted, and His_6_-tagged Rep proteins were affinity-purified using Co^2+^ resin and analyzed by western blotting. Slower-migrating species of both Rep1 (Fig. 2A, bottom panel, lane 1) and Rep2 (Fig. 2B, bottom panel, lane 1) were detected that had mobilities consistent with these being sumoylated forms. To determine whether these species were SUMO-conjugates of Rep1 and Rep2, the proteins were expressed in yeast over-expressing either untagged or HA epitope-tagged SUMO (HA-SUMO). As expected, the major slower-migrating forms of Rep1 and Rep2 displayed lower mobility when SUMO was HA-tagged (bottom panels of Fig. 2A and 2B, lane 5), a shift consistent with the additional molecular weight of the three added HA epitopes. These species were verified to be HA-SUMO conjugates by their reactivity with an anti-HA antibody (top panels in Fig. 2A and 2B, lane 5). As expected for HA-SUMO conjugates of Rep1 and Rep2, respectively, these species were not detected by the anti-HA antibody in metal-ion affinity-purified protein extracted from yeast that did not express Rep1 or Rep2 (Fig. 2A and 2B, lane 7). Notably, the lysine-to-arginine mutations in Rep1_3R_ and Rep2_13R_ that impaired two-hybrid interaction with SUMO also reduced the levels of the HA-tagged SUMO-conjugates of Rep1 and Rep2 (top panels in Fig. 2A and 2B, lane 6), suggesting that Rep1_3R_ and Rep2_13R_ are sumoylation-deficient mutants.

In addition to the slower-migrating forms of Rep1 and Rep2 that were reduced in abundance for the Rep1_3R_ and Rep2_13R_ mutants, a species of Rep1 migrating at ∼100 kDa was also observed (Fig. 2A, bottom panel, lane 1). We do not know the nature of this species. The species co-migrates with one of the high-molecular weight Rep1-HA-SUMO-conjugates detected by the anti-HA antibody (Fig. 2A, top panel, lane 5) but was observed even when all Rep1 lysine residues with the exception of the three most carboxy-terminal were mutated to arginine and was not consistently reduced in abundance by the mutations in Rep1_3R_ (data not shown). We speculate that this species might represent a post-translational modification to Rep1 other than sumoylation.

### Lysine-to-arginine substitutions in Rep1_3R_ and Rep2_13R_ that impair SUMO conjugation also perturb 2 µm plasmid inheritance

**Figure 3:**
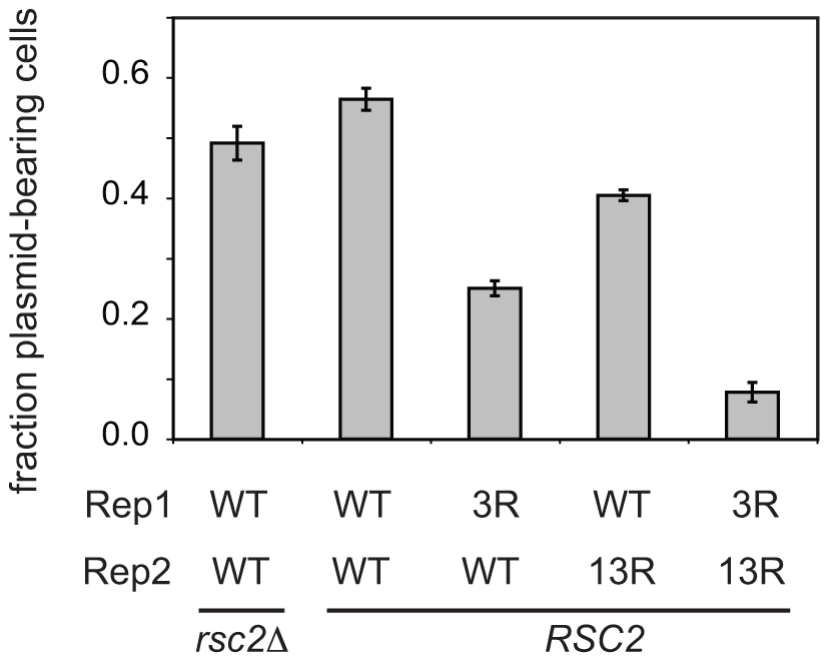
Mutations that inhibit sumoylation of Rep1 and Rep2 also impair plasmid maintenance. Yeast lacking the native 2 µm plasmid were transformed with *KanMX6*-tagged 2 µm plasmids encoding the indicated Rep1 and Rep2 alleles, grown in medium containing G418 and the proportion of plasmid-containing (G418-resistant) cells (mean ± SEM from six independent yeast transformants) was determined.

To investigate whether the mutations in Rep1_3R_ and Rep2_13R_ affect the function of the Rep proteins in partitioning of the 2 µm plasmid, we compared the inheritance of 2 µm-based plasmids encoding either wild-type Rep proteins or the Rep1_3R_ and Rep2_13R_ mutants. Since the native 2 µm plasmid confers no phenotype, we introduced the *KanMX6* gene that confers resistance to the aminoglycoside antibiotic G418 [50] into the plasmids. The *FLP* gene was inactivated in these plasmids so that defects in plasmid partitioning would not be obscured by the copy-number amplification activity of Flp. To examine inheritance of the *KanMX6*-tagged 2 µm plasmids encoding wild-type or mutant Rep1 and Rep2, the plasmids were introduced into *cir* ^0^ yeast. The fraction of cells in the population containing plasmid was measured after ∼15 generations of growth in the presence of G418, a condition under which only cells that have inherited plasmid can continue to proliferate. Under this selective pressure, the fraction of plasmid-free cells in the population should remain consistently low as plasmid-containing cells out-compete plasmid-free cells. However, if plasmid partitioning is sufficiently impaired, daughter cells will more frequently receive no copies of the high copy number plasmid, and the proportion of G418-sensitive cells in the population will be increased. As a control for the assay, inheritance of the *KanMX6*-tagged 2 µm plasmid encoding wild-type Rep proteins was monitored in *cir^0^* yeast lacking the *RSC2* gene (*rsc2Δ). RSC2* encodes a regulatory subunit of one form of the RSC chromatin remodeling complex [51], and has previously been shown to be required for maintenance of the native 2 µm plasmid [28]. In the assay used here, the fraction of G418-resistant cells in the *rsc2Δ* cell population (0.49 ±.02) was reduced compared to that of *RSC2* yeast (0.56 ±.03) (Fig. 3). This is consistent with a measured loss rate of 8.0% (± 1.2) of plasmid-containing cells per generation for the *KanMX6*-tagged 2 µm plasmid in *rsc2Δ* cells when cultured in the absence of G418 (data not shown). When the inheritance of the *KanMX6*-tagged 2 µm plasmid encoding lysine-to-arginine mutant versions of Rep1 and Rep2 was assayed in wild-type *cir^o^* yeast, the fraction of plasmid-containing cells was significantly lower than when Rep1 and Rep2 were wild type, with the amino acid substitutions in Rep1 having a more significant impact on plasmid inheritance than those in Rep2 (Fig. 3). The reduction in the proportion of cells containing the plasmid when only Rep2 was mutant was similar to that observed for *rsc2Δ* yeast containing the *KanMX6*-tagged 2 µm plasmid encoding wild type Rep1 and Rep2. When both Rep1 and Rep2 were mutant, the fraction of plasmid-containing cells was reduced further still, indicating that an even higher proportion of daughter cells was consistently receiving no copies of the plasmid during cell division. The more severe inheritance defect caused by the lysine-to-arginine mutations in Rep1_3R_ and Rep2_13R_ relative to that caused by the chromosomal *rsc2Δ* mutation known to cause spontaneous loss of the endogenous 2 µm plasmid suggests that a native 2 µm plasmid with these mutations would also be lost at high frequency.

### Mutations in Rep1_3R_ and Rep2_13R_ do not alter their post-translational stability, or the ability of Rep2 to chaperone Rep1

**Figure 4:**
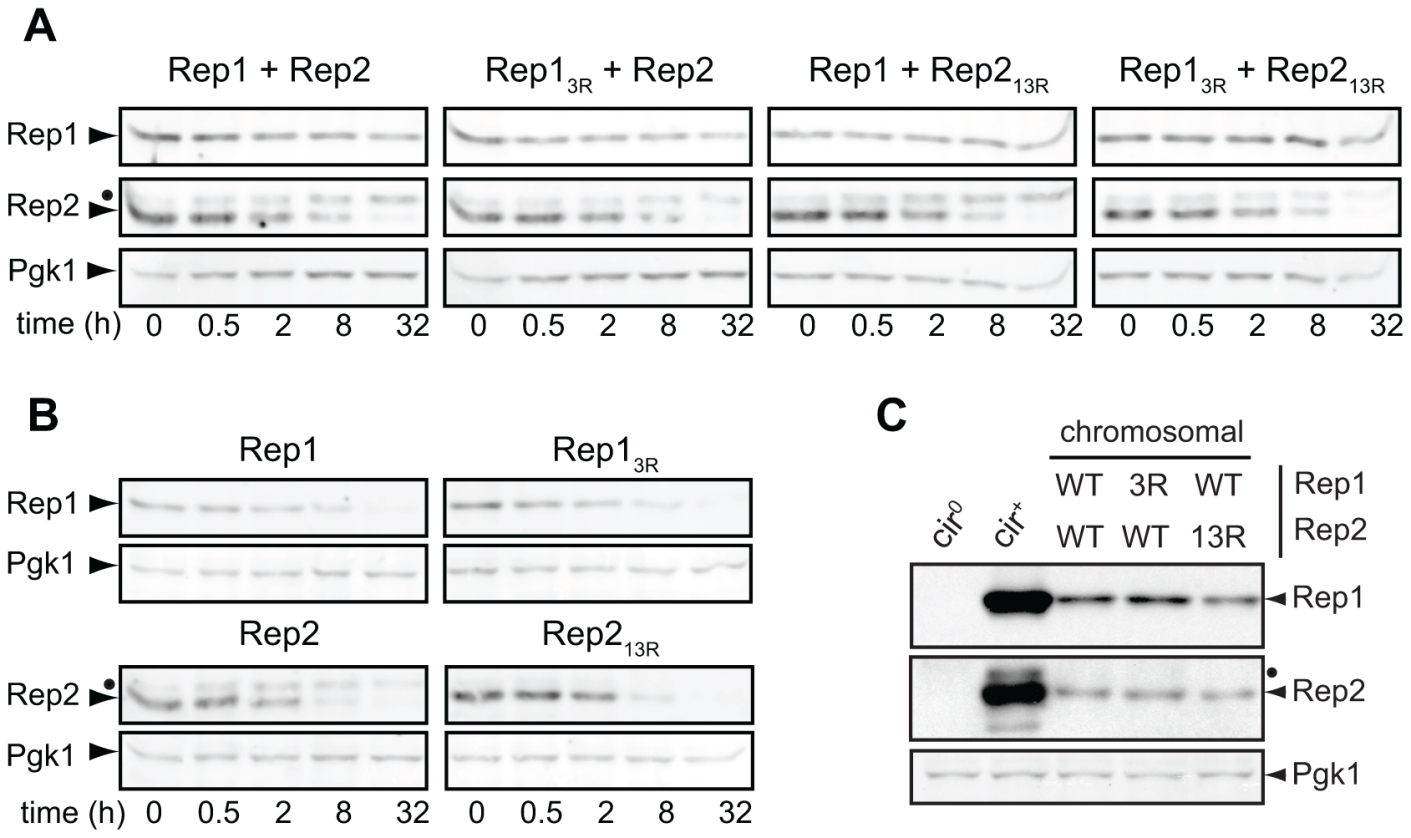
Effects of lysine-to-arginine mutations in Rep1 and Rep2 on post-translational stability. Rep1 and Rep2 were (A) co-expressed or (B) expressed in the absence of one another and protein was extracted from yeast at the indicated time points following addition of cycloheximide. A closed circle indicates a phosphorylated species of Rep2 (unpublished data). (C) Yeast with the indicated alleles of *REP1* and *REP2* integrated in the genome were cultured and protein extracted. Rep protein levels were examined by western blot analysis.

The defect in 2 µm plasmid maintenance associated with the Rep1_3R_ and Rep2_13R_ mutants could be due to altered steady-state levels of the mutant Rep proteins. Mutation of lysine residues in the Rep proteins could alter their half-life if sumoylation of these sites contributes to Rep1 and Rep2 post-translational stability. Alternatively, the lysine residues might normally be directly targeted for ubiquitin-mediated proteolysis, or mutation of these residues could significantly perturb protein folding or other post-translational modifications of the Rep proteins. To examine Rep1 and Rep2 post-translational stability, *cir* ^0^ yeast were transformed with single-copy (*CEN*/*ARS*) plasmids encoding *REP1* or *REP2* under control of a galactose-inducible promoter. Expression from non-2 µm-based plasmids using a heterologous promoter ensured that Rep1 and Rep2 protein levels would not be affected by differences in plasmid copy number caused by mis-segregation, or altered Rep protein-dependent regulation of *REP* gene expression [52], [53]. The transformed yeast were transferred to medium containing galactose to activate *GAL1* promoter-driven expression of the *REP* genes. After a brief period sufficient for Rep protein expression, cycloheximide was added to inhibit translation, and Rep1 and Rep2 protein levels were monitored by western blotting. The levels of a highly stable glycolytic enzyme, 3-phosphoglycerate kinase (Pgk1) [54], were simultaneously examined (Fig. 4A). We did not observe any significant difference in the stability of wild-type Rep1 and Rep2 compared to their respective sumoylation-deficient mutants. However, we did notice that Rep1 had a much shorter half-life when expressed in the absence of Rep2 (Fig. 4B), suggesting that interaction of Rep2 with Rep1 contributes to Rep1 post-translational stability. We have previously observed that point mutations in Rep1 that abolish association with Rep2 produce a similar reduction in Rep1 steady-state levels (unpublished data), consistent with Rep2 association protecting Rep1 from degradation.

Although mutation of the sites required for sumoylation did not reduce the half-life of the Rep proteins expressed from the *GAL1* promoter, the high level of expression might have masked proteolytic turnover that would alter steady-state levels if expressed from their native promoters. Comparison of the levels of wild-type versus sumoylation-deficient Rep proteins expressed from 2 µm-based plasmids was precluded by differences in plasmid copy number resulting from defective partitioning of plasmids encoding Rep1_3R_ and Rep2_13R_ (data not shown). To circumvent the plasmid copy number differences, the *REP1* and *REP2* genes or their lysine-to-arginine mutant derivatives were integrated into the chromosome in a *cir* ^0^ yeast strain, and steady-state Rep protein levels were examined by western blotting (Fig. 4C). The level of Rep1_3R_ did not significantly differ from wild-type Rep1, indicating that the lysine substitutions did not affect Rep1 protein stability, and suggesting that no critical ubiquitination sites were destroyed in the process of eliminating Rep1 SUMO attachment sites. A slight reduction in the level of both Rep1 and Rep2 was observed when Rep2 was mutant. Since the *REP* genes were expressed from their own promoters, and both *REP* genes are regulated by Rep1 and Rep2 [52], [53], reduced Rep protein levels could indicate a slight reduction in transcription of both *REP* genes in the presence of Rep2_13R_. Alternatively, the slightly lower level of both proteins could indicate increased turnover of the mutant Rep2_13R_ protein, which would result in less Rep2 being available to stabilize Rep1.

### The Rep1_3R_ and Rep2_13R_ mutants are defective for association with the plasmid-partitioning locus

**Figure 5:**
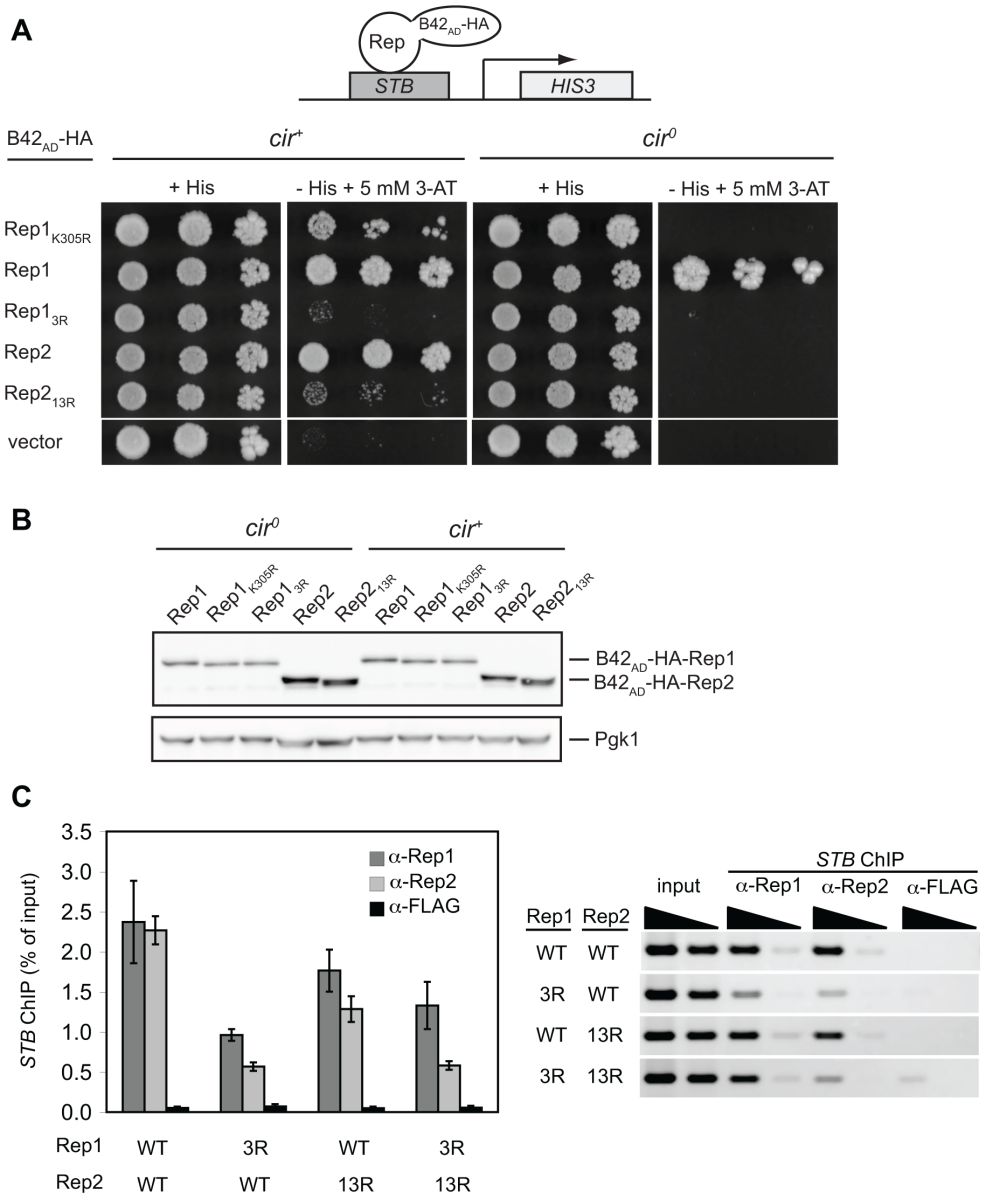
Lysine mutations in Rep1_3R_ and Rep2_13R_ impair Rep-*STB* association but do not reduce levels of Rep fusion proteins. (**A**) Five-fold serial dilutions of a *cir* ^+^ or *cir* ^0^ yeast one-hybrid reporter strain encoding *STB* upstream of a *HIS3* reporter gene and transformed with plasmids encoding the indicated Rep1 and Rep2 proteins fused to B42_AD_-HA were spotted onto galactose media to induce expression of fusion proteins. Recruitment of the Rep proteins to *STB* was monitored by growth on medium lacking histidine supplemented with 5 mM 3-aminotriazole. (B) Levels of the B42_AD_-HA fusion proteins were monitored by western blot analysis of total protein extracted from the yeast transformants 24 h after galactose induction. (C) ChIP assays were performed with anti-Rep1, anti-Rep2, or anti-FLAG antibodies and the precipitated DNA amplified using primers specific for *STB*. ChIP efficiency is indicated by the percent of input DNA immunoprecipitated (avg ± sd from triplicate assays) (left) and ethidium-stained agarose gels of PCR products from a representative assay are shown (right). Template DNA amplified in “input” PCR reactions represented 40% of the DNA that was immunoprecipitated and used as template in “ChIP” PCR reactions.

Point mutations in Rep1 that prevent its recruitment to the 2 µm plasmid *STB* locus have previously been shown to result in plasmid mis-segregation [24]. To examine the ability of Rep1_3R_ and Rep2_13R_ to associate with *STB in vivo*, we used a one-hybrid assay. Rep proteins were expressed as HA epitope-tagged, viral B42 activation domain (B42_AD_-HA) fusion proteins in a yeast strain in which the plasmid *STB* locus was integrated in the genome upstream of a histidine biosynthetic reporter gene (*HIS3)*. In this assay, expression of *HIS3* is dependent on interaction of the respective Rep fusion protein with *STB* and can be assessed by monitoring growth of the yeast on medium lacking histidine and supplemented with 3-aminotriazole (3-AT), a competitive inhibitor of the *HIS3* gene product [55] (Fig. 5A). Yeast expressing B42_AD_-HA alone (vector) exhibited no growth, indicating that the viral activation domain cannot be recruited to *STB* by itself. As previously reported, in a *cir* ^+^ reporter strain, expression of wild-type Rep1 or Rep2 fused to B42_AD_-HA led to robust growth, indicating that both Rep proteins are recruited to *STB* [55]. The single K305R point mutation in Rep1 that only slightly diminished its interaction with SUMO in a two-hybrid assay modestly reduced one-hybrid *STB* association, while the mutations in Rep1_3R_ and Rep2_13R_ that virtually abolished two-hybrid interaction with SUMO severely impaired their ability to associate with *STB* in the one-hybrid assay. We also examined the ability of the Rep proteins to associate with *STB* in an isogenic *cir* ^0^ strain where any contribution to the interaction from endogenous plasmid proteins would be lost (Fig. 5A). The difference in *STB* interaction between wild-type Rep1 and the two sumoylation-deficient Rep1 mutants was more pronounced in the *cir* ^0^ reporter strain. The Rep1-K305R mutant expressed as a B42_AD_-HA fusion protein did not result in growth of the reporter strain, suggesting that loss of this single sumoylation site was sufficient to significantly reduce association of Rep1 with the *STB* locus in the absence of native plasmid proteins. Expression of native Rep1 or Rep2 individually from a second plasmid in the *cir* ^0^ reporter strain also expressing B42_AD_-HA-Rep1_K305R_ or B42_AD_-HA-Rep1_3R_ did not result in any growth, suggesting that the combined presence of endogenous Rep1 and Rep2 proteins was required for the limited association of the sumoylation-deficient Rep1 mutants with *STB* observed in the *cir* ^+^ reporter strain (data not shown). Interestingly, although wild-type Rep2 expressed as a B42_AD_-HA fusion protein could activate the *STB*-driven *HIS3* reporter gene in the *cir* ^+^ strain, no growth was observed for the *cir* ^0^ reporter strain, suggesting B42_AD_-HA-Rep2 was dependent on endogenous plasmid proteins for association with *STB*.

A reduction in the steady-state levels of the mutant Rep1 and Rep2 fusion proteins could also explain the reduced one-hybrid association with *STB*. However, no significant differences in Rep fusion protein levels in the *cir* ^+^ or *cir* ^0^ reporter strains were observed (Fig. 5B), suggesting that reduced activation of the reporter gene by the mutant Rep fusion proteins was a consequence of reduced interaction with the *STB* sequence in the promoter rather than being due to lower abundance.

To determine whether Rep1_3R_ and Rep2_13R_ were impaired for interaction with *STB* in a native context, the efficiency of chromatin immunoprecipitation (ChIP) of the *STB* locus with anti-Rep1 or anti-Rep2 antibodies was examined for 2 µm-based plasmids encoding wild-type or mutant Rep proteins (Fig. 5C). Consistent with results of the one-hybrid assays, *STB* DNA co-immunoprecipitated less efficiently when the plasmid encoded Rep1_3R_ or Rep2_13R_ rather than wild-type Rep1 and Rep2. Taken together, the results of the one-hybrid and ChIP assays indicate that defects in the inheritance of plasmids encoding Rep1_3R_ and Rep2_13R_ may be due to impaired interaction of the mutant Rep proteins with the plasmid partitioning locus. *STB* was also less efficiently co-immunoprecipitated with anti-Rep2 antibodies in yeast expressing Rep1_3R_, suggesting that association of Rep2 with *STB* is, in part, dependent on the stable association of Rep1 with *STB*. In contrast, *STB* was efficiently co-immunoprecipitated by anti-Rep1 antibodies when the plasmid encoded wild-type Rep1 and the Rep2_13R_ mutant, suggesting Rep1 association with *STB* was not affected by the mutations in Rep2_13R_.

### Rep2 depends on Rep1 for robust association with *STB*

**Figure 6:**
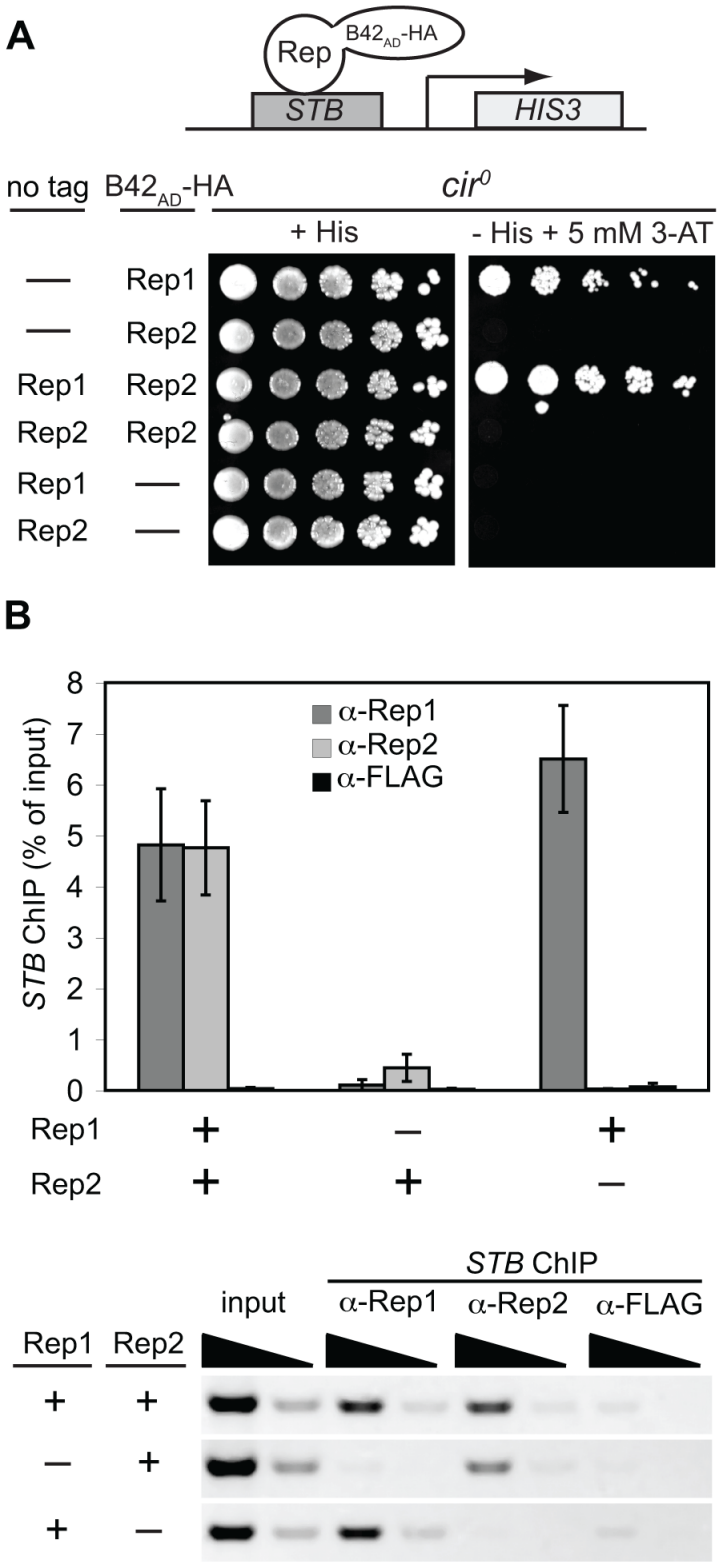
Stable association of Rep2 with *STB* depends on Rep1. (A) A *cir* ^0^ yeast one-hybrid reporter strain was co-transformed with single-copy plasmids allowing for galactose-inducible expression of untagged and B42_AD_-HA-tagged Rep1 or Rep2. Expression of the *STB*-driven *HIS3* reporter gene was monitored by growth on solid galactose media as described in the legend to Fig. 5. (B) ChIP assays were performed on extracts from yeast expressing Rep1, Rep2, or both Rep1 and Rep2 from an *ADE2*-tagged 2 µm plasmid as detailed in the legend to Fig. 5.

The impaired association of Rep2 with *STB* when Rep1 was absent (Fig. 5A) or when Rep1 interaction with *STB* was reduced due the lysine mutations in Rep1_3R_ (Fig. 5C) suggested that Rep2 may depend on interaction with Rep1 for stable association with *STB*. Although previous work has shown that Rep2, when over-expressed, is able to interact with *STB* in the absence of Rep1 [6], the degree to which Rep1 and Rep2 associate with *STB* in the absence of the partner protein has not been examined. To investigate this, we assessed the interaction of B42_AD_-HA-Rep2 with *STB* in a *cir* ^0^ one-hybrid reporter strain in which either untagged Rep1 or untagged Rep2 was expressed from the *GAL1* promoter (Fig. 6A). In this assay, expression of Rep1, but not Rep2, promoted association of B42_AD_-HA-Rep2 with *STB*. To examine the dependence of Rep1 and Rep2 on each other for their association with *STB* in a native context, we performed ChIP assays using yeast transformed with a marker-tagged 2 µm plasmid encoding Rep1 and Rep2, or derivatives that lacked either the *REP1* or *REP2* gene (Fig. 6B). While *STB* did co-immunoprecipitate with anti-Rep2 antibodies when Rep1 was absent, the yield was significantly lower than when Rep1 was present. In contrast, absence of Rep2 did not reduce association of Rep1 with *STB*. These results suggest that *STB*-bound Rep1 may promote more stable association of Rep2 with the plasmid-partitioning locus.

### Mutations in Rep1_3R_ cause loss of normal Rep1 punctate nuclear staining pattern

**Figure 7:**
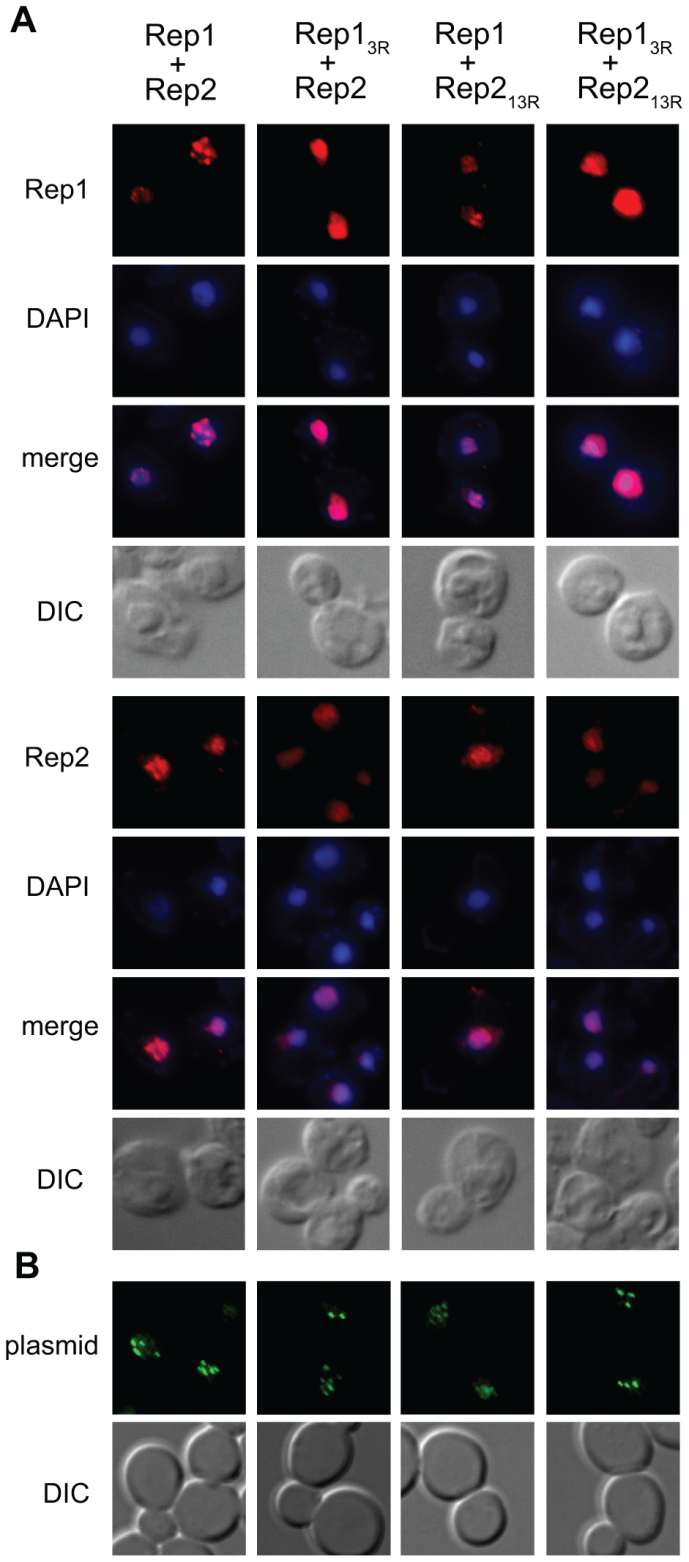
Localization of Rep1_3R_ and Rep2_13R_. (A) Spheroplasts were prepared from *cir* ^0^ yeast expressing wild-type or mutant Rep1 and Rep2 from an *ADE2*-tagged 2 µm plasmid and the Rep proteins were visualized by indirect immunofluorescence. Bulk chromatin was visualized by DAPI staining and cells by light microscopy (DIC). (B) Yeast were co-transformed with an *ADE2*-tagged 2 µm plasmid encoding the indicated Rep1 and Rep2 alleles and a plasmid containing the *STB* locus and 256 *lacO* repeats. Plasmid localization was visualized by fluorescence microscopy following induction of GFP-LacI repressor fusion protein expression.

The multiple copies of the 2 µm plasmid are organized in a small number of nuclear foci that co-localize with Rep1 and Rep2 [5], [27]. In *rsc2Δ* yeast, impaired association of Rep1 with *STB* [24] is accompanied by loss of the normal Rep1 punctate nuclear staining pattern [28]. The impaired interaction of Rep1_3R_ and Rep2_13R_ with the plasmid *STB* locus observed in the one-hybrid and ChIP assays suggested that localization to the plasmid foci might be affected by these mutations. To assess this, we used indirect immunofluorescence to compare localization of wild-type Rep proteins and the Rep1_3R_ and Rep2_13R_ mutants expressed from marker-tagged 2 µm plasmids (Fig. 7A). As previously reported, wild-type Rep1 was present in distinct nuclear foci [5], [27], [56] (Fig. 7A, column 1); however, Rep1_3R_ showed diffuse nuclear staining (Fig. 7A, columns 2 and 4). The absence of Rep1 foci when the protein had lysine substitutions that impaired sumoylation was consistent with the results of the one-hybrid and ChIP assays and suggests either that sumoylation is required for Rep1 localization to the distinct sub-nuclear plasmid-containing domains or that association with the plasmid *STB* locus is required for Rep protein sumoylation. Wild-type Rep2, when co-expressed with wild-type Rep1, exhibited a less sharply punctate but still uneven nuclear staining pattern as has been previously reported [5], [56] (Fig. 7A, column 3). However, Rep2 expressed with Rep1_3R_ displayed a more uniform, pan-nuclear staining pattern (Fig. 7A, column 2), consistent with results of ChIP assays that demonstrated that association of Rep2 with *STB* was impaired when Rep2 was co-expressed with Rep1_3R_ (Fig. 5C). When Rep2_13R_ was expressed with wild-type Rep1 (Fig. 7A, column 3), the Rep2 punctate staining pattern was similar to that of wild-type Rep2, suggesting Rep2 is not dependent on being sumoylated for its nuclear distribution. The respective staining patterns of Rep1 and Rep2 when both were mutant (Fig. 7A, column 4) were similar to those observed when only Rep1 was mutant.

The mislocalization of Rep1 and Rep2 when Rep1 was sumoylation-deficient prompted us to examine plasmid localization in the presence of Rep1_3R_ and Rep2_13R_ mutants. To visualize 2 µm plasmid foci, we introduced a 2 µm reporter plasmid containing 256 lac operator sequences in yeast that expressed a GFP-tagged LacI repressor protein (Fig. 7B) [5]. As has been previously reported, the plasmid was localized into a small number of distinct nuclear foci in cells expressing wild-type Rep1 and Rep2 [5], [27]. These foci were still observed when both Rep proteins were mutant, suggesting that clustering of plasmid copies is not dependent on Rep protein sumoylation.

### Rep1 with an I202T substitution loses association with the *STB* locus but interacts with SUMO in a two-hybrid assay

**Figure 8:**
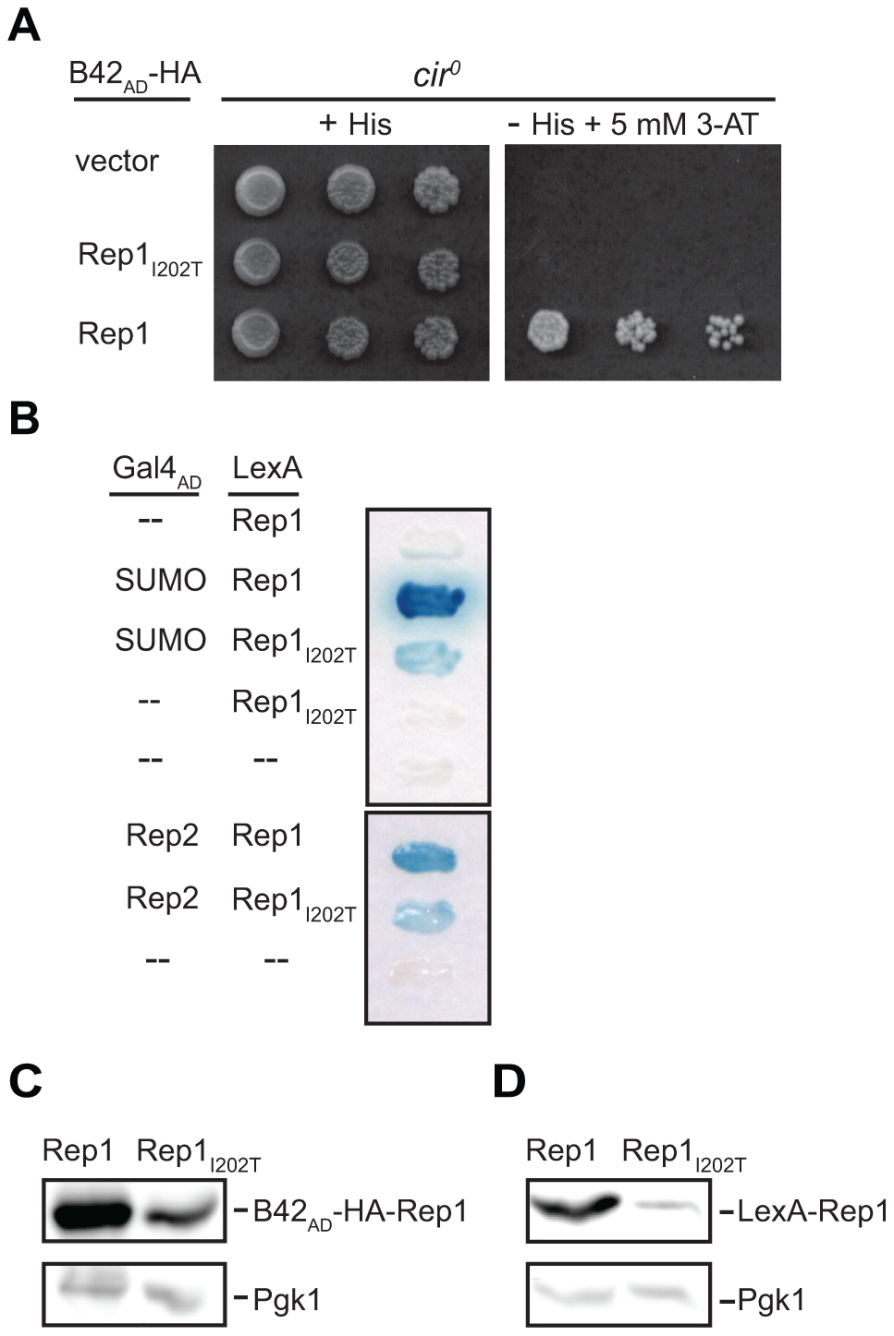
Rep1_I202T_ does not associate with the plasmid *STB* locus but retains interaction with SUMO. (A) Association of wild type and I202T mutant Rep1 (Rep1_I202T_) with *STB* in *cir^0^* yeast was monitored using a one-hybrid assay as described in the legend to Figure 5. (B) Interaction of Rep1 and Rep1_I202T_ with SUMO and with Rep2 was monitored in *cir^0^* yeast using a two-hybrid assay as described in the legend to Figure 1. Levels of the Rep1 and Rep1_I202T_ fusion proteins in the (C) one-hybrid and (D) two-hybrid reporter yeast strains, in A and B respectively, were monitored by western blotting analysis with antibodies specific for Pgk1 (C and D, bottom), anti-HA (C, top) and anti-LexA (D, top).

Our data suggest that the lysine-to-arginine mutations in Rep1_3R_ and Rep2_13R_ impair their ability to be sumoylated and their interaction with *STB*, implying that for both Rep1 and Rep2, sumoylation status correlates with the ability to associate with *STB*. Sumoylation of Rep1 and Rep2 may be required for their stable association with *STB*. Alternatively, association with *STB* may promote sumoylation of Rep1 and Rep2. To attempt to distinguish between these two possibilities, we made use of a mutant *REP1* allele isolated in a screen for mutations in *REP1* that impaired plasmid partitioning (unpublished data). The mutant Rep1 has threonine substituted for isoleucine at residue 202 (Rep1_I202T_), and was chosen because the altered residue lies outside the domain required for Rep1 self-association and Rep2 interaction [36], [55], was not within the region containing the three lysine-to-arginine substitutions in Rep1_3R_, and leads to loss of association of Rep1 with *STB* in a one-hybrid assay in both *cir^+^* and *cir^0^* strains (unpublished data and Fig. 8A). To determine whether this mutant version of Rep1 could be sumoylated despite loss of *STB* association, Rep1_I202T_ was assessed for interaction with SUMO in a two-hybrid assay (Fig. 8B). Interaction between Rep1_I202T_ and SUMO was observed although expression of the β-galactosidase reporter gene was weaker than when Rep1 was wild type (Fig. 8B). A similar reduction in the expression of the reporter gene was also observed for interaction of Rep1_I202T_ with Rep2 when compared to interaction of wild-type Rep1 with Rep2 (Fig. 8B). The similar degree of reduction for both types of interaction suggested that the steady-state level of the Rep1_I202T_ fusion protein might be lower that that of the wild type Rep1 fusion. Western blotting analysis confirmed reduced steady-state levels for the Rep1_I202T_ fusion proteins in both the one-hybrid (Fig. 8C) and two-hybrid reporter strains (Fig. 8D). Weaker activation of the reporter gene by the mutant Rep1_I202T_ in the two-hybrid assays would be expected from the reduced level of the fusion protein (Fig. 8D) and does not necessarily represent impaired interaction of Rep1_I202T_ with SUMO or Rep2. While the complete loss of detectable *STB*-association for Rep1_I202T_ in the one-hybrid assay might be due to the reduced level of the fusion protein, the observed loss of association of Rep1_I202T_ with *STB* is not sufficient to abolish interaction of Rep1_I202T_ with SUMO in the two-hybrid assay. This result is consistent with our observations that Rep1 and Rep2 both retain two-hybrid interaction with SUMO in yeast lacking an *STB* sequence (data not shown).

## Discussion

Equal partitioning of the multiple copies of the yeast 2 µm plasmid at mitosis is dependent on the association of plasmid proteins Rep1 and Rep2 with each other, and with the plasmid-partitioning locus, *STB* [5], [36], [55]–[58]. Here we have identified distinct roles for Rep1 and Rep2 in this process; Rep1 stabilizes Rep2 association with *STB,* while Rep2 association with Rep1 increases Rep1 post-translational stability. We have demonstrated that like Flp, the 2 µm-encoded site-specific recombinase required for plasmid copy number amplification, and Rep2 [11], Rep1 is sumoylated, and that lysine-to-arginine mutations in Rep1 and Rep2 that inhibit their sumoylation also impair their association with *STB* and lead to defective plasmid partitioning.

Both Rep1 and Rep2 required simultaneous mutation of multiple lysine residues to effectively block their sumoylation (Rep1_3R_ and Rep2_13R_ mutants, respectively). Based on two-hybrid assays, the three sites mutated in Rep1_3R_ each likely represent sites targeted for SUMO conjugation; however, it seems unlikely that the thirteen residues mutated in Rep2_13R_ each normally serve as a SUMO acceptor site. Proteins targeted for sumoylation often contain multiple SUMO acceptor sites [59], but in other studies, abolishing major sumoylation sites has been shown to lead to increased SUMO conjugation at less preferred sites [60], [61] and this may also be the case for Rep1 and Rep2. Despite the multiple mutations in Rep1_3R_ and Rep2_13R_ that abolished two-hybrid interaction with SUMO, faint slower-migrating Rep protein species were detected in western blot analysis, suggesting sumoylation was still occurring, albeit at a low level, on remaining lysine residues. However, since inheritance of 2 µm plasmids encoding Rep1_3R_ or Rep2_13R_ mutants was perturbed, the residual sumoylation was not sufficient for normal Rep protein function.

Sumoylation can have diverse effects, altering the activity, interactions, localization or stability of a targeted protein [14]. Our analyses demonstrated that Rep1 and Rep2 were not dependent on sumoylation for their interaction with each other, consistent with observations that bacterially-expressed Rep1 and Rep2 interact *in vitro* [36], [56], [57]. However, the Rep1_3R_ and Rep2_13R_ mutants were defective for association with the plasmid *STB* partitioning locus. Even mutation of a single sumoylation site in Rep1, K305, modestly reduced Rep1-*STB* association. Since efficient partitioning of the 2 µm plasmid has been shown to depend on association of Rep1 and Rep2 with *STB* [24], loss of association of Rep1 and Rep2 lysine-to-arginine mutants with *STB* is likely to be the primary defect leading to loss of efficient plasmid partitioning.

While the Rep1_3R_ and Rep2_13R_ mutants were both impaired for association with *STB*, the mutations in Rep1_3R_ led to more severe defects in maintenance of a 2 µm plasmid compared to those in Rep2_13R_. This result was somewhat unexpected in light of observations that absence of either Rep1 or Rep2 results in an equally severe defect in plasmid partitioning [58], [62], [63]. However, ChIP assays also supported this difference, showing that the amount of *STB* DNA that co-immunoprecipitated with Rep2 was markedly reduced when Rep2 was expressed with Rep1_3R_ or in the absence of Rep1, while Rep1 association with *STB* was not dependent on the presence of Rep2. Our results, combined with those from earlier studies [24], [36], [64] suggest that Rep2 may recognize *STB* in the absence of Rep1, but this limited association is inadequate for function. The increased sensitivity of *STB* chromatin to micrococcal nuclease in yeast lacking Rep1, or both Rep1 and Rep2, but not when only Rep2 was absent [65], supports the hypothesis that Rep1 is either more directly or more tightly associated with *STB* than Rep2. Co-evolution of *STB* and Rep1 sequence variants in 2 µm plasmids isolated from several laboratory and industrial *S. cerevisiae* strains [66], [67] also supports a model in which Rep1 recognition of *STB* is a critical prerequisite to productive association of Rep2 with *STB.*

Although Rep1 association may be an essential first stage in establishing the plasmid-partitioning complex, Rep2 association with *STB* also correlated with plasmid inheritance. Mutations that led to loss of Rep1 sumoylation perturbed both Rep2-*STB* association and plasmid partitioning more severely than mutations associated with loss of Rep2 sumoylation. The milder defect in plasmid partitioning associated with the Rep2_13R_ mutant suggests that Rep2_13R_, recruited to *STB* through its interaction with wild-type Rep1, can confer some partitioning function. Although the partitioning defect produced by the lysine-to-arginine substitutions in Rep2_13R_ was mild compared to when Rep1_3R_ was expressed, it was similar to that observed when the RSC2 chromatin remodeling complex was impaired, a defect severe enough to make yeast unable to maintain the native 2 µm plasmid [28]. We also identified another key role for Rep2 in this process, namely the stabilization of the Rep1 protein. This chaperoning activity of Rep2 may explain why loss of Rep2 leads to an equally severe defect in plasmid inheritance as loss of Rep1 [58], [62], [63]. A reduction in Rep1 levels when Rep2 is absent and a requirement for Rep1 to stabilize Rep2 association with *STB* would explain why absence of either would result in a similar failure to recruit critical host proteins to *STB* [6], [8], [9], [24].

A hierarchical assembly of Rep protein sub-complexes has previously been proposed to mediate early events required for establishing the functional 2 µm plasmid-partitioning complex. Recruitment of the motor protein Kip1 to *STB* was shown to be dependent on both Rep1 and Rep2, but Kip1 was found to co-immunoprecipitate only with Rep2, and not with Rep1 [8]. Downstream events, including recruitment of the centromere-specific histone H3 variant Cse4, the RSC2 complex, and ultimately the cohesin complex [6], [8], [9], [24], [28] are dependent on Kip1 recruitment to *STB* [8].

Our data indicate a correlation between sumoylation status of the Rep proteins and their ability to associate with *STB*. Is sumoylation of Rep1 and Rep2 required for association with *STB*, or does recruitment to *STB* lead to their sumoylation? For the replication clamp protein, PCNA, sumoylation has been shown to be dependent on PCNA association with DNA [68]. In *Xenopus*, sumoylation of centromere-associated proteins is dependent on their co-localization with SUMO E3 ligase PIASy at centromeres of mitotic chromosomes [69]. Therefore, for the yeast plasmid Rep proteins we cannot exclude the possibility that the lysine substitutions in Rep1_3R_ and Rep2_13R_ directly impair interactions with *STB* DNA or with host proteins in a manner that consequently leads to reduced sumoylation of the Rep proteins. Rep1 has not been shown to have intrinsic DNA-binding activity, precluding *in vitro* assessment of the effect of the lysine substitutions on DNA association. However, the observation that a Rep1 mutant (Rep1_I202T_) impaired for *in vivo* association with *STB* could still interact with SUMO in a two-hybrid assay supports a model in which sumoylation promotes an early stage in the assembly of the Rep partitioning complex, enabling stable association of the Rep proteins at *STB*. SUMO-regulated association with DNA has been demonstrated for heat shock proteins Hsf1 and Hsf2, which are dependent on sumoylation for their DNA-binding activity [70], [71].

Consistent with the hypothesis that impaired sumoylation of Rep1 correlates with defective assembly of the Rep1-Rep2 complex at *STB*, when Rep1 was sumoylation-deficient, both Rep proteins lost their localization to discrete nuclear foci previously shown to contain clusters of the 2 µm plasmid [5], [27], [56]. The uniform nuclear staining pattern observed for sumoylation-deficient Rep1 is reminiscent of that observed for wild-type Rep1 in *rsc2Δ* yeast [28], in which association of Rep1 with *STB* is also impaired [24], supporting Rep1 interaction with *STB* as critical for Rep1 sub-nuclear localization. Sumoylation has been shown to regulate the sub-nuclear localization of other proteins, a notable example being the SUMO-dependent localization of promyelocytic leukemia (PML) protein to PML nuclear bodies in mammalian cells [72]. In this study, 2 µm plasmid foci were observed even when Rep1 and Rep2 were both sumoylation-deficient. Further investigation is needed to assess whether localization of plasmid clusters in their normal spindle pole-proximal nuclear address [5], [7] is altered by changes in Rep1 and Rep2 sumoylation status.

Our data suggest that Rep protein sumoylation may promote stable association of the Rep proteins with 2 µm plasmid DNA, an association reminiscent of sumoylation-dependent targeting of proteins to centromeres in yeast and in higher eukaryotes. In human cells, proteins conjugated with SUMO-2/3 are enriched at centromeres [73]. In yeast, topoisomerase II is robustly targeted to pericentric DNA when translationally fused to SUMO [74], and yeast kinetochore proteins Ndc10, Cep3, Bir1, and Ndc80 are all SUMO targets [75], with sumoylation of Ndc10 being functionally relevant. Sumoylation-deficient Ndc10 fails to localize to the mitotic spindle, resulting in defective chromosome segregation [75]. The short defined point centromeres to which Ndc10 binds are unique to the Saccharomycetaceae family of budding yeast, and were recently proposed to have arisen by replacement of a typical epigenetic fungal centromere with an ancestral 2 µm plasmid-derived partitioning system [76]. While rapid evolution may have obscured sequence homology between the 2 µm plasmid and chromosomal segregation proteins, it is tempting to speculate that post-translational modification of segregation proteins with SUMO might be a conserved process, essential for their common function.

The yeast 2 µm plasmid is not the only parasitic DNA element to exploit the host cell SUMO pathway for its maintenance [77]. Many viral proteins involved in maintenance of the episomes that encode them are SUMO-modified. Members of the human papillomavirus E2 family of proteins are dependent on sumoylation for their ability to tether viral genomes to host chromosomes to ensure faithful segregation [78]. Host sumoylation has therefore frequently been exploited to ensure maintenance of parasitic genomes in eukaryotic cells, and here we have presented evidence that suggests the yeast 2 µm plasmid may also co-opt this essential cellular process to ensure its efficient segregation during host cell division.
